# Discriminating Valid from Spurious Indices of Phase-Amplitude Coupling

**DOI:** 10.1523/ENEURO.0334-16.2016

**Published:** 2017-01-16

**Authors:** Ole Jensen, Eelke Spaak, Hyojin Park

**Affiliations:** 1 Centre for Human Brain Health, School of Psychology, University of Birmingham, Birmingham B15 2TT, United Kingdom; 2 Department of Experimental Psychology, University of Oxford, OX1 3UD Oxford, United Kingdom; 3 Institute of Neuroscience and Psychology, University of Glasgow, Glasgow G12 8QB, United Kingdom

**Keywords:** alpha, EEG, gamma, MEG, oscillations, theta

## Abstract

Recently there has been a strong interest in cross-frequency coupling, the interaction between neuronal oscillations in different frequency bands. In particular, measures quantifying the coupling between the phase of slow oscillations and the amplitude of fast oscillations have been applied to a wide range of data recorded from animals and humans. Some of the measures applied to detect phase-amplitude coupling have been criticized for being sensitive to nonsinusoidal properties of the oscillations and thus spuriously indicate the presence of coupling. While such instances of spurious identification of coupling have been observed, in this commentary we give concrete examples illustrating cases when the identification of cross-frequency coupling can be trusted. These examples are based on control analyses and empirical observations rather than signal-processing tools. Finally, we provide concrete advice on how to determine when measures of phase-amplitude coupling can be considered trustworthy.

## Significance Statement

Neuronal oscillations at different frequencies are thought to reflect processing within and across brain networks. To fully understand how these oscillations support neuronal computation, it is essential to understand how they interact. It is, however, not straightforward to quantify cross-frequency interactions. We here discuss the problems associated with quantifying cross-frequency coupling and put forward examples in which indices of cross-frequency interactions can be considered reliable.

## Detecting phase-amplitude coupling

**Figure 1.**
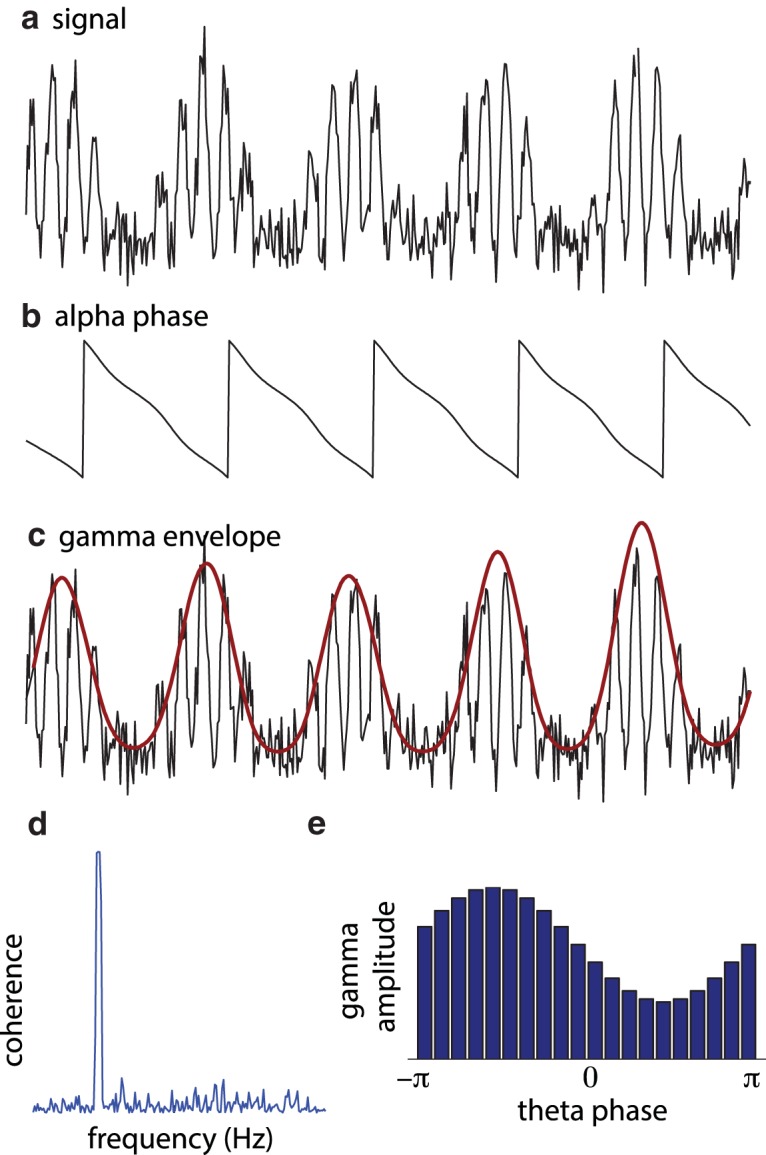
Quantifying PAC: ***a***, The raw signal. ***b***, ***c***, The temporal evolution of phase (***b***) and power (***c***) are typically identified using a discrete Fourier transform, wavelet transformations, or a bandpass filter followed by a Hilbert transformation. ***d***, ***e***, The relationship between phase and amplitude envelope can be quantified by the phase relationship between the two signals (e.g., coherence; ***d***; Osipova et al., 2008) or by considering the nonuniformity of the phase distribution of fast-frequency power with respect to the slow oscillations (***e***; Tort et al., 2010).

In the past decade, there has been an increasing interest in the role of brain oscillations in both human and animal research (Buzsáki, 2006). Since these oscillations co-occur in different frequency bands, their functional role cannot be understood in isolation, and it is imperative to uncover how they interact. There are different ways by which these oscillations can interact [e.g., phase-to-phase, amplitude-to-amplitude, and phase-to-amplitude coupling (PAC); Palva et al., 2005; Jensen and Colgin, 2007; Siegel et al., 2012]. In particular, PAC has received strong interest since the phenomenon suggests that the phase of slow oscillations correlates with neuronal activity in higher-frequency bands (Bragin et al., 1995; Canolty et al., 2006; Tort et al., 2009; Miller et al., 2010; Belluscio et al., 2012; Lisman and Jensen, 2013; von Nicolai et al., 2014; Bonnefond and Jensen, 2015; Colgin, 2015; Florin and Baillet, 2015; Hyafil et al., 2015a,b; McLelland and VanRullen, 2016; Fig. 1). Such findings have provided important insights into the temporal coordination of neuronal activity. However, the reliability of PAC has recently been questioned since the measure is sensitive to nonsinusoidal properties of the neuronal oscillations (Kramer et al., 2008; Aru et al., 2015). Indeed, recent articles have reported PAC, which can fully be explained by the lower-frequency oscillation, as having a saw-tooth like shape (Cole et al., 2016; Sheremet et al., 2016; Cole and Voytek, 2017). Although these concerns are valid, they do not exclude the existence of a true measure of PAC that is associated with neuronal activity in different frequency bands. The aim of this commentary is to provide examples for cases where measures of PAC can be considered trustworthy. We will conclude by providing concrete recommendations on how to determine whether measures of PAC should be considered trustworthy.

**Figure 2.**
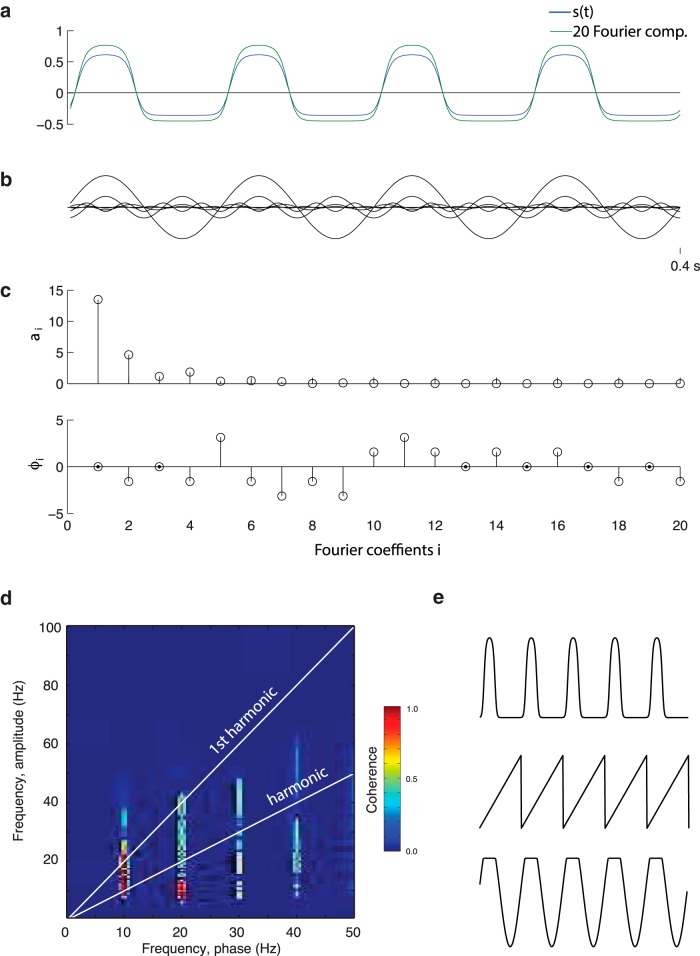
Periodic nonsinusoidal signals expressed as Fourier series. ***a***, A periodic nonsinusoidal signal (blue line) constructed by a sigmoidal function applied to sinusoidal signal: $s\left(t\right)={f(0.5+0.5\sin\left(2\pi 10t\right))}^{}\;where\;f\left(x\right)=\;\frac{1}{\left(1+\;e^{-12(x-0.7)}\right)}$. The signal can be expressed as a sum of sinusoids $s\left(t\right)=\;\alpha_{1}\;sin(2\pi ft+\varphi_{1)}+\;\alpha_{2}\sin\left(2\pi \left(2f\right)t+\varphi_{2}\right)+\;\alpha_{3}\sin\left(2\pi \left(3f\right)t+\varphi_{3}\right)\ldots$. i.e. the periodic signal can be expressed as a sum of sinusoids at the harmonic frequencies. The green line shows the sum of the first 10 harmonics. ***b***, The first 10 harmonics. ***c***, The coefficient for amplitude and phase (*α_i_* and $\varphi_{i}$). ***d***, The PAC for the signal is sensitive to the higher harmonics and can therefore produce a spurious phase-to-power coupling. ***e***, Examples of nonsinusoidal functions of some neuronal relevance: periodic pulses, a saw-tooth function, and a clipped sinusoidal signal.

**Figure 3.**
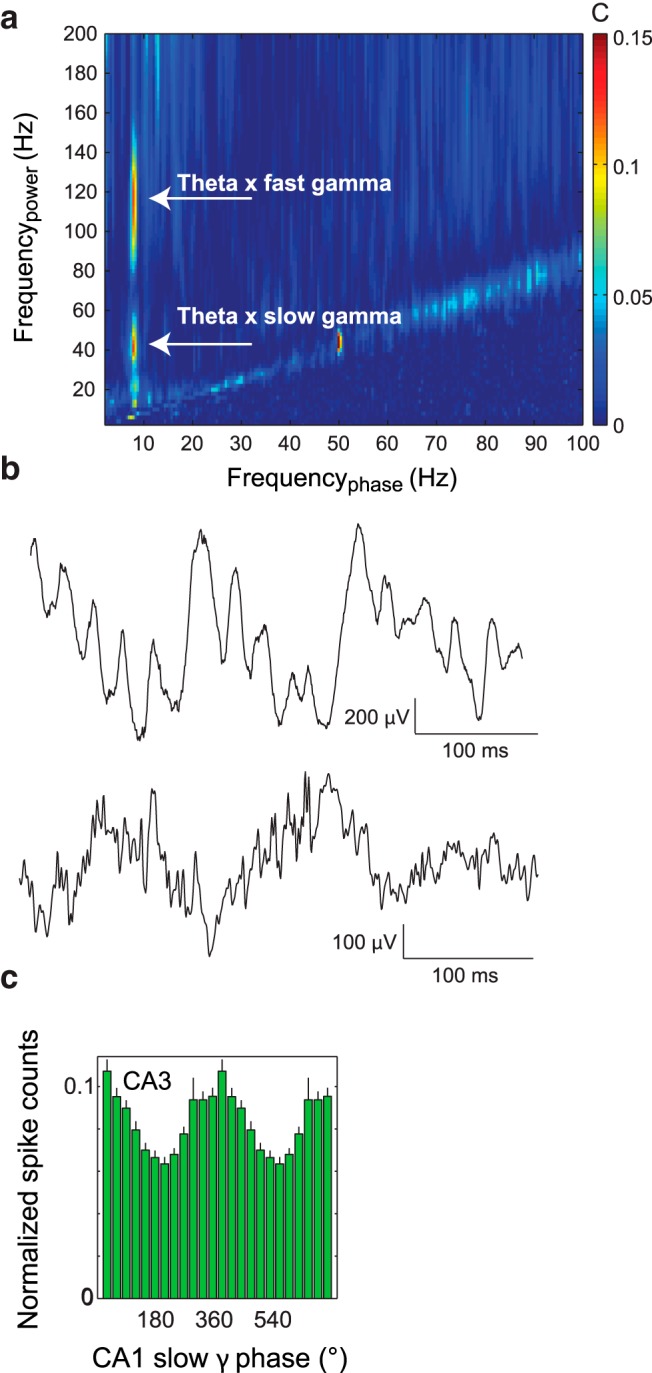
Gamma oscillations are modulated by theta phase in the rat hippocampus. ***a***, The PAC measure applied to the LFP of rat data reveals phase-amplitude coupling. ***b***, Gamma oscillations coupled to the phase of the theta oscillations are directly visible in the unfiltered LFPs. ***c***, A histogram demonstrating the neuronal firing is modulated by gamma band oscillations. Reprinted by permission from Macmillan Publishers Ltd. (Colgin et al., 2009).

Even though there are several metrics by which PAC can be quantified, these different methods are qualitatively similar. The core of these methods is that they (1) estimate the phase of the slow oscillations (Fig. 1*b*), (2) estimate the temporal evolution of amplitude or power (the “envelope”) of the faster signal (Fig. 1*c*), and (3) relate the phase of the slow oscillations to the time course of power of the faster signal (Fig. 1*d*,*e*).

The time course of phase and power (points 1 and 2) can be estimated by a sliding time window subjected to a discrete Fourier transformation, a wavelet transformation (typically, a Morlet wavelet), or a bandpass filter followed by a Hilbert transformation. These three approaches, at some level, all involve a Fourier-like transformation multiplying the signal by $e^{-i2\pi ft}$, where *f* is the frequency of interest (Bruns, 2004). The slow and fast signals (point 3) are related by estimating the phase synchronization (e.g., coherence) between the slow signal and the envelope of the fast signal (Osipova et al., 2008; Fig. 1*d*) or by quantifying the distribution of power of the fast signal with respect to the phase of the slow signal (Canolty et al., 2006; Tort et al., 2010; Fig. 1*e*). Strong coherence or a nonuniform phase distribution of high-frequency power is indicative of PAC. Another method includes a general linear model approach to quantify the relationship between phase and amplitude (van Wijk et al., 2015). All these measures have in common that they are sensitive to coupling in the sense of stronger high-frequency amplitude at certain low-frequency phases than at others. When applying these PAC measures, a broad set of frequency ranges are typically explored, yielding a frequency-by-frequency measure of the coupling.

## The problem: spurious identification PAC coupling

The goal of reporting PAC is to identify coupling in which amplitude in the high-frequency band is associated with actual neuronal activity in that frequency band, which is modulated by slow neural oscillations. However, the amplitude modulations at fast frequencies might not be caused by neuronal firing in that frequency range per se but could also stem from higher harmonics generated by the oscillations in the slow-frequency band. From standard Fourier analysis (Stein and Shakarchi, 2003), it follows that any periodic signal repeated at frequency *f*_0_ can be expressed as a sum of sinusoidal functions with frequencies *f*_0_, 2*f*_0_, 3*f*_0_, … (Fig. 2). Importantly, the more the periodic signal deviates from a sinusoidal function, the larger the coefficients of the higher harmonics. Any nonsinusoidal neural oscillation will therefore necessarily have power in higher harmonics. Importantly, depending on the exact waveform shape, the application of a technique like a sliding time window Fourier transform (or equivalent) will result in this higher harmonic power showing a modulation as a function of phase of the slow oscillation. The higher harmonics are not reflecting “true neuronal activity” per se, as they may not be associated with spiking or oscillations at these frequencies. Measures of PAC are sensitive to higher harmonics, as shown in Figure 2*d*. Note that, due to frequency smoothing as a result of estimating the envelope for the fast frequencies, the harmonic contributions are often “bleeding” together in the PAC plot. Therefore, great care must be taken when interpreting measures of PAC, in particular in the frequency bands of the higher harmonics, additionally taking into account the effective frequency smoothing (Kramer et al., 2008; Aru et al., 2015; Cole et al., 2016; Jones, 2016; Lozano-Soldevilla et al., 2016). In particular, Cole et al. (2016) and Sheremet et al. (2016) provide concrete examples from respective intracranial human and hippocampal rat recordings in which nonsinusoidal oscillations contribute to estimates of PAC.

## How are nonsinusoidal oscillations generated?

What will cause neuronal oscillations to be nonsinusoidal? One possibility is a gradual ramping up of neuronal activity within a slow cycle. This would produce a saw-tooth like shape (Fig. 2*e*), which indeed has been observed in the rat hippocampus (Terrazas et al., 2005; Belluscio et al., 2012; Sheremet et al., 2016) and in electrocorticography recordings in the human motor cortex (Cole et al., 2016). Another possibility is pulses of neuronal activity repeated at a fixed frequency. Yet another possibility is “clipping” in which the periodic signal is capped when exceeding a certain magnitude. This could be explained by periodic neuronal activity “maxing out” (e.g., due to a depletion of some resource). While clipping is a theoretical possibility, we are not aware of reports of such effects. As described, nonsinusoidal wave shapes will produce higher harmonics, resulting in spurious identification of PAC. As with many measures of neural activity, one must be cautious of potential confounds like these when interpreting measures of PAC. We would here like to emphasize that the possibility of spurious measures of coupling by no means precludes the existence of true PAC associated with neuronal activity in various frequency bands.

## Three examples of reliable phase-to-amplitude coupling

How best to handle the concerns of spurious identification of PAC? Although advanced signal-processing techniques are being developed to alleviate problems with spurious coupling (Dvorak and Fenton, 2014; van Driel et al., 2015; Cole et al., 2016; Soto et al., 2016), it is problematic to implement signal-processing tools that unequivocally remove the effects of higher harmonics when calculating the PAC measure. However, even when signal-processing tools cannot provide a conclusive answer, there are specific empirical circumstances that might alleviate concerns about the spurious identification of coupling. We will here provide three such examples.

### Example 1: neuronal spiking clocked by gamma oscillations

The core concern in PAC is that activity at higher frequencies is caused by harmonics rather than neuronal firing at higher frequencies. Intracranial animal recordings allow us to discern both spikes and local field potentials (LFPs). Such recording is routinely performed in behaving rats using electrodes implanted in the hippocampus. Such recordings have revealed that hippocampal activity in the gamma band is modulated by the phase of the theta oscillations (Bragin et al., 1995; Colgin et al., 2009; Belluscio et al., 2012; Fig. 3*a*). Given that hippocampal theta oscillations are clearly nonsinusoidal (Belluscio et al., 2012), higher harmonics are indeed a concern. However, Colgin et al. (2009) were able to identify reliable gamma oscillations directly visible in the LFP (Fig. 3*b*). Interestingly, both fast and slow gamma oscillations were identified. Furthermore, analysis of spike-triggered LFP recordings revealed that spike timing was clocked by the phase of ongoing gamma oscillations (Fig. 3*c*). These findings provide direct evidence for actual neuronal activity with a dominant gamma frequency component. By simultaneously considering neuronal spiking and LFPs, we can confidently conclude that the coupling between gamma activity and theta phase reported in the rat hippocampus is due to genuine oscillatory activity in the gamma band.

### Example 2: slow and fast oscillations are generated by different populations

**Figure 4.**
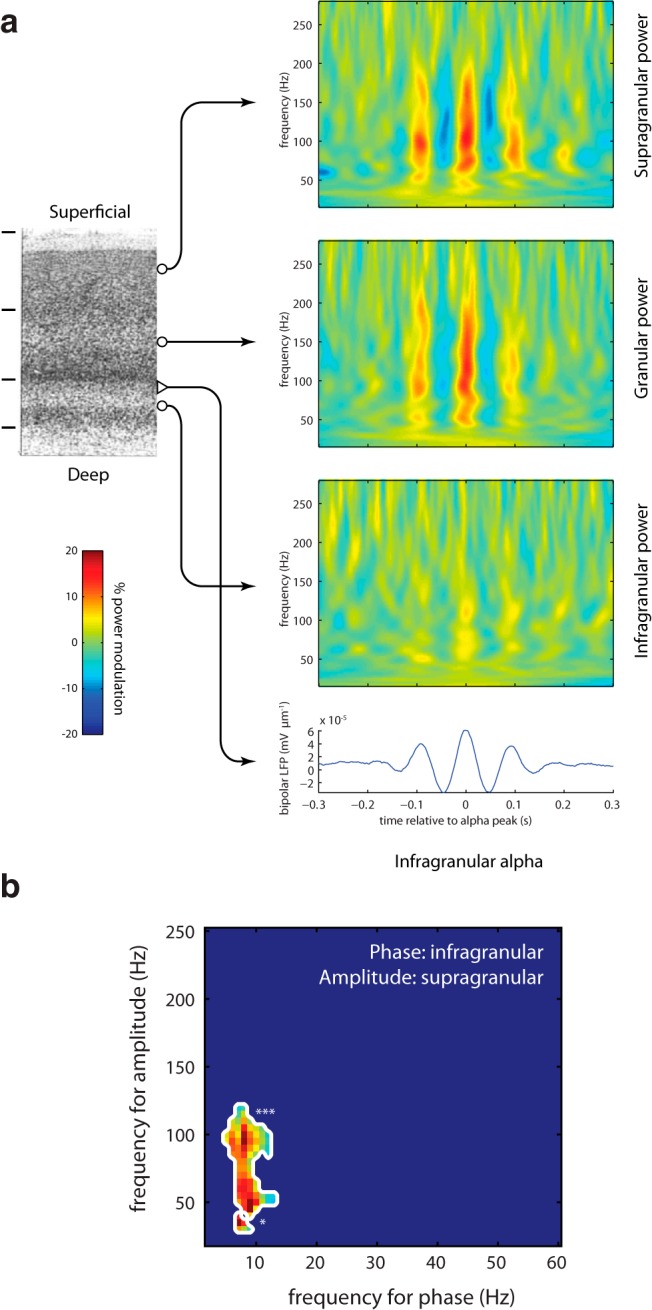
Gamma power in superficial cortical layers is coupled to alpha phase in deeper layers. ***a***, Epochs of data in the alpha range are phase aligned according to the peaks in each epoch. For each epoch, the time–frequency representation of power is calculated and averaged. These data were obtained from laminar recordings in visual cortex of the monkey. Note that coupling was stronger across sites than within sites. This would not have been the case if the coupling was explained by nonsinusoidal alpha oscillations. ***b***, The PAC measure applied to the data from the deep and superficial layers. Reproduced with permission from Cell Press (Spaak et al., 2012).

PAC is not constrained to data from single site recordings. It can also be applied to quantify the relationship between the phase of oscillations in one site and fast activity in another site (van der Meij et al. 2012). This was done in the study by Spaak et al. (2012) in which data from laminar recordings in visual cortex of the monkey were analyzed. The core finding was that the phase of alpha oscillations in the deeper layers (infragranular layers) was coupled to the gamma band activity in superficial layers (supragranular layers; Fig. 4). Importantly, the PAC between layers was stronger than the local PAC in any of the layers. This finding provides evidence for different populations generating the alpha and gamma band activity. The observation strengthens the case that gamma activity coupled to alpha phase is not spurious. Had the effect been due to higher harmonics, it would have been stronger locally than across sites. In conclusion, measures of PAC in which the slow and fast activity are generated in distinct layers, areas, or populations reduce concerns on spurious coupling.

### Example 3: coupling increases when low-frequency power decreases

**Figure 5.**
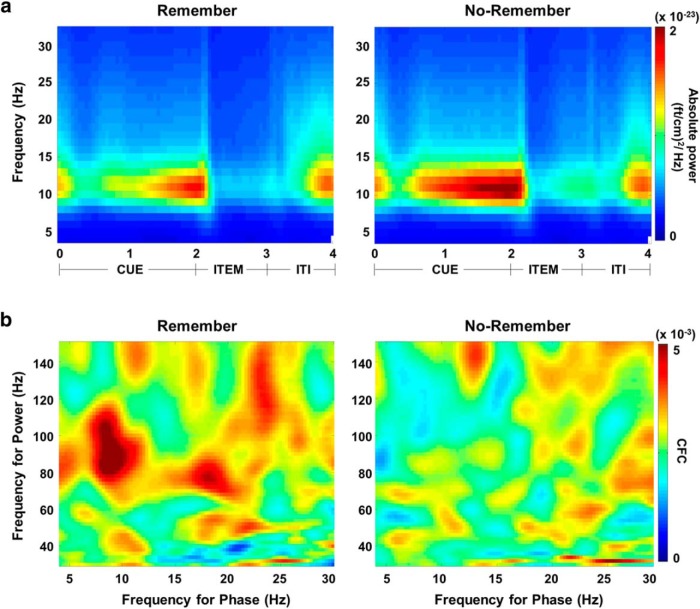
An MEG study in which subjects were asked to encode (‘Remember’) or ignore (‘No-Remember’) upcoming visual stimulus. The ‘Remember’ condition was associated with less alpha power (***a***) but stronger PAC (***b***). Given that PAC goes up when alpha power goes down, the coupling is not likely to be explained by higher harmonics of the alpha oscillations. Reproduced with permission from Macmillan Publishers Ltd. (Park et al., 2016).

A recent memory study relying on human MEG investigated PAC when subjects were preparing to encode upcoming visual items (Park et al., 2014, 2016). When subjects were asked to ignore the upcoming item (“No-Remember”), alpha power in visual cortex was stronger compared with when subjects were asked to remember (“Remember”) the item (Fig. 5*a*). However, importantly, in the Remember condition, when alpha power was low, the PAC coupling was stronger (Fig. 5*b*). This finding is consistent with the notion that alpha oscillations inhibit gamma band activity in a phasic manner (Mazaheri and Jensen, 2008). The stronger the alpha oscillations, the lower the gamma power and therefore the weaker the PAC in the No-Remember condition. Had the PAC been explained by the nonsinusoidal shape of the alpha oscillations, and thus by the higher harmonics of the alpha frequency, one would expect PAC to increase with alpha power since the magnitude of the higher harmonics would increase as well. Park et al. (2016) found the reverse, in support of nonspurious PAC. It does remain a theoretical possibility that low-amplitude alpha oscillations are less sinusoidal than high-amplitude alpha oscillations. However, this is not likely to explain the effect on PAC, since the lower amplitude would also impair the alpha phase estimate and thereby reduce the PAC. In sum, studies in which one can demonstrate an increase in PAC associated with a decrease in power speak to the existence of nonspurious coupling.

## Does broadband gamma activity warrant special caution?

Transient power in higher harmonics at certain phases of a lower-frequency oscillation, together with frequency smoothing due to power estimation, might result in a typical broadband frequency response at those low-frequency phases that contain sharp transitions (e.g., the peak of a saw tooth). One should be extra careful about such broadband PAC profiles. However, even the occurrence of broadband (“gamma”) activity modulated by slow oscillations does not per se imply that the measured PAC is caused by nonsinusoidal slow oscillations. As we will explain below, the broadband response could be caused by neuronal spiking, providing a wide frequency content. Indeed, it is debated whether neuronal activity in the gamma band can be considered oscillatory or whether it is a broadband phenomenon (Hermes et al., 2015). This issue is complicated by factors such as differences between species, tasks, brain regions, and recording techniques. For instance, Colgin et al. (2009) have reported fast and slow gamma oscillations, both in a well defined, relatively narrow frequency band, in the behaving rat. These were isolated in spike-field recordings and were associated with, respectively, retrospective and prospective memory operations (Bieri et al., 2014). In contrast, several other studies report broadband activity modulated by slow oscillations (Canolty et al., 2006). The latter should, however, not be considered spurious PAC. Broadband activity modulated by a slow rhythm does speak to the temporal organization of neuronal computation since it demonstrates that the neuronal activity is synchronized more strongly at some phases than others within a cycle of a slow rhythm. This phasic synchronization is bound to modulate the communication to target regions and is likely to be important for neuronal communication (Jensen, 2005; Hyafil et al., 2015b; McLelland and VanRullen, 2016).

## Conclusion

Nonsinusoidal low-frequency waveforms will have transient power at higher harmonics in certain phases of the cycle. When analyzing PAC in such signals, one might erroneously conclude that higher-frequency neural activity is clocked by a lower-frequency one. Even advanced signal-processing tools might never completely alleviate this potential artifact. Instead, one can look to other empirical factors to aid the interpretation of PAC. We have here presented three examples of reports of PAC that cannot be explained by concerns such as higher harmonics. While great care should be taken when interpreting PAC measures, there are numerous good examples of true coupling between distinct neuronal activity at slow and fast frequencies. Although there is no magic bullet, we recommend searching for complementary evidence, as follows:

- Accumulate evidence from complementary modalities. For instance, human data recorded by EEG and MEG can be related to intracranial electrocorticographic recordings from patients. Use spike-field data in rodents or monkeys to elucidate whether the gamma activity in question can be associated with the coordination of neural firing.
- Investigate coupling between cortical layers or regions. For instance, if alpha or theta phase drives gamma power in a neighboring region more strongly than within the phase-providing region itself, concerns on spurious PAC are reduced.
- Relate modulations in low-frequency power to modulations in PAC. A lack of correlation or a negative correlation makes the interpretation as a harmonic artifact less likely.

We encourage a continued debate on when measures of PAC can be considered to reflect neuronal coupling between oscillations and fast neuronal activity. Given careful interpretation, we believe PAC measures are an essential tool for understanding the temporal organization of hierarchical neuronal computation.

## Synthesis

Reviewing Editor: Bradley Postle, University of Wisconsin Decisions are customarily a result of the Reviewing Editor and the peer reviewers coming together and discussing their recommendations until a consensus is reached. When revisions are invited, a fact-based synthesis statement explaining their decision and outlining what is needed to prepare a revision will be listed below. The following reviewer(s) agreed to reveal their identity: Michael Cohen, Bradley Voytek

The two reviews have both found your revision to be appreciably improved over the original:

Reviewer 1: "The revision is improved. I still think the title does not really fit. I would have called it something like "Case studies of true neural phase-amplitude coupling". But I'll leave that up to the authors and editor to decide."

Reviewer 2: "The authors have addressed all of my concerns. This piece is a nice commentary on a quickly-moving area of systems neuroscience."

I am in agreement with Reviewer 1, and will request that you consider a making a few stylistic modifications to the title and to the body of your commentary, that I think will sharpen and more precisely articulate the points that you want to make. With regard to the title, R2 and I both think that the formulation "when to trust" doesn't succinctly convey the arguments, nor the contents of your paper. My suggestion would be "Discriminating valid from spurious indices of phase-amplitude coupling". Getting into the commentary itself, I would encourage you to be careful to distinguish the physical phenomenon of PAC from analytic measures that are intended to index this phenomenon. Thus, for example, in the Abstract, you presumably don't intend to mean that non-sinusoidal properties the oscillations literally "create spurious coupling", but rather that they can some measures to 'spuriously indicate the presence of coupling'. And in the following sentence, rather than "While such spurious coupling has been observed, in this commentary we give concrete examples illustrating cases when cross-frequency coupling can be trusted", I think what you intend to say is that while such instances of 'the spurious identification of coupling' have been observed, you illustrate cases in which the identification of CFC can be trusted. At the end of the Significance Statement, what you are doing is putting forward examples in which 'indices of' cross-frequency interactions can be considered reliable. I won't go on through the entirety of the manuscript, but I hope can see how being careful to distinguish the measure of the phenomenon from the phenomenon itself with help clarify your arguments.

Finally, one detail that I note in your revision is that since the time that you first submitted this manuscript to eNeuro, the manuscript that you have been citing as "Cole SR, Peterson EJ, van der Meij R, de Hemptinnee C, Starr PA, Voytek B (2016). Nonsinusoidal oscillations underlie pathological phase-amplitude coupling in the motor cortex in Parkinson's disease. BioRxiv." has now been accepted at a peer-reviewed journal, as so should be updated to: "Cole SR & Voytek B. Brain oscillations and the importance of waveform shape. Trends Cogn Sci". If you'd like to see a preprint, it's available at http://voyteklab.com/publications/
